# 4,6-Bis[5-methyl-3-(trifluoro­meth­yl)pyrazol-1-yl]pyrimidine

**DOI:** 10.1107/S1600536809043657

**Published:** 2009-10-28

**Authors:** Yong-Hong Li, Tao Zhang, Xiang-Dong Mei, Jun Ning

**Affiliations:** a Key Laboratory of Pesticide Chemistry and Applications, Ministry of Agriculture, Institute of Plant Protection Academy of Agricultural Sciences, Beijing 100193, People’s Republic of China

## Abstract

The complete mol­ecule of the the title compound, C_14_H_10_F_6_N_6_, is generated by crystallographic twofold symmetry, with two C atoms lying on the roatation axis. The dihedral angle between the central and peripheral rings is 25.97 (8)°.

## Related literature

For background to fluorine-containing heterocycles and their properties, see: Krishnaiah & Narsaiah (2002 ▶); Ohno *et al.* (2004 ▶).
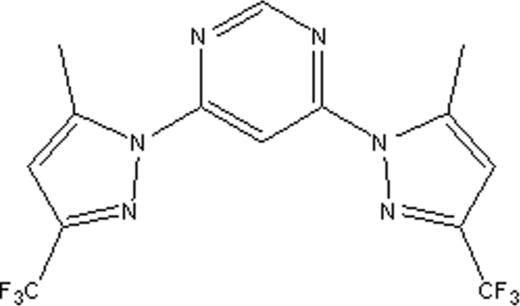


## Experimental

### 

#### Crystal data

C_14_H_10_F_6_N_6_
*M* *_r_* = 376.28
Monoclinic, 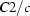

*a* = 8.5387 (14) Å
*b* = 16.110 (6) Å
*c* = 11.022 (5) Å
β = 99.295 (5)°
*V* = 1496.2 (9) Å^3^
*Z* = 4
Mo *K*α radiation
μ = 0.16 mm^−1^
*T* = 173 K
0.41 × 0.36 × 0.26 mm

#### Data collection

Rigaku Saturn724+ CCD diffractometer
Absorption correction: multi-scan (*CrystalClear*; Rigaku, 2008 ▶) *T*_min_ = 0.938, *T*_max_ = 0.960
8915 measured reflections
1706 independent reflections
1678 reflections with *I* > 2σ(*I*)
*R*_int_ = 0.033

#### Refinement

*R*[*F* ^2^ > 2σ(*F* ^2^)] = 0.047
*wR*(*F* ^2^) = 0.100
*S* = 1.19
1706 reflections
120 parameters
H-atom parameters constrained
Δρ_max_ = 0.23 e Å^−3^
Δρ_min_ = −0.22 e Å^−3^

### 

Data collection: *CrystalClear* (Rigaku, 2008 ▶); cell refinement: *CrystalClear*; data reduction: *CrystalClear*; program(s) used to solve structure: *SHELXS97* (Sheldrick, 2008 ▶); program(s) used to refine structure: *SHELXL97* (Sheldrick, 2008 ▶); molecular graphics: *SHELXTL* (Sheldrick, 2008 ▶); software used to prepare material for publication: *SHELXL97*.

## Supplementary Material

Crystal structure: contains datablocks I, global. DOI: 10.1107/S1600536809043657/hb5156sup1.cif

Structure factors: contains datablocks I. DOI: 10.1107/S1600536809043657/hb5156Isup2.hkl

Additional supplementary materials:  crystallographic information; 3D view; checkCIF report

## Figures and Tables

**Fig. 1.**
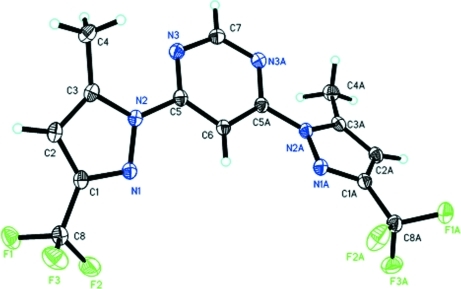
The molecular structure of the title compound showing 50% probability displacement ellipsoids and atom-numbering scheme.

## supplementary crystallographic information

### Comment

Strategically positioned fluorine in heterocyclic compounds, especially those containing trifluromethyl groups plays an important role in medicines and agrochemicals (e.g. Krishnaiah & Narsaiah, 2002). Specifically, the fluorinated pyrazoles have been shown to possess high biological activities (e.g. Ohno *et al.* 2004) as herbicides, fungicides, insecticides, analgesics, antipyretics and antiinflammatories. Pyrazolopyrimidine and related fuesd heterocycles are of interest as potential bioactive molecules. Recently, the new title compound (I) was synthesized in our group with high herbicidal activity. The crystal structure of the compound (I) is shown in Fig. 1.

### Experimental

The title compound (0.1 g) was dissolved in anhydrous methanol (20 ml) at room temperature. Colourless blocks of (I) were obtained through slow evaporation after two weeks.

### Refinement

All the hydrogen atoms were placed in idealised positions with C—H = 0.93–0.98Å and *U*_iso _(H) = 1.2–1.5*U*_ep_ (C).

### Crystal data

**Table:**

C_14_H_10_F_6_N_6_	*F*(000) = 760
*M**_r_* = 376.28	*D*_x_ = 1.670 Mg m^−^^3^
Monoclinic, *C*2/*c*	Mo *K*α radiation, λ = 0.71073 Å
Hall symbol: -C 2yc	Cell parameters from 648 reflections
*a* = 8.5387 (14) Å	θ = 2.2–27.5°
*b* = 16.110 (6) Å	µ = 0.16 mm^−^^1^
*c* = 11.022 (5) Å	*T* = 173 K
β = 99.295 (5)°	Block, colorless
*V* = 1496.2 (9) Å^3^	0.41 × 0.36 × 0.26 mm
*Z* = 4	

### Data collection

**Table:**

Rigaku Saturn724+ CCD diffractometer	1706 independent reflections
Radiation source: sealed tube	1678 reflections with *I* > 2σ(*I*)
graphite	*R*_int_ = 0.033
ω scans at fixed χ = 45°	θ_max_ = 27.5°, θ_min_ = 2.7°
Absorption correction: multi-scan (*CrystalClear*; Rigaku, 2008)	*h* = −11→11
*T*_min_ = 0.938, *T*_max_ = 0.960	*k* = −20→20
8915 measured reflections	*l* = −14→14

### Refinement

**Table:**

Refinement on *F*^2^	Primary atom site location: structure-invariant direct methods
Least-squares matrix: full	Secondary atom site location: difference Fourier map
*R*[*F*^2^ > 2σ(*F*^2^)] = 0.047	Hydrogen site location: inferred from neighbouring sites
*wR*(*F*^2^) = 0.100	H-atom parameters constrained
*S* = 1.19	*w* = 1/[σ^2^(*F*_o_^2^) + (0.0291*P*)^2^ + 1.4916*P*] where *P* = (*F*_o_^2^ + 2*F*_c_^2^)/3
1706 reflections	(Δ/σ)_max_ < 0.001
120 parameters	Δρ_max_ = 0.23 e Å^−^^3^
0 restraints	Δρ_min_ = −0.22 e Å^−^^3^

### Special details

**Table:**

Geometry. All e.s.d.'s (except the e.s.d. in the dihedral angle between two l.s. planes) are estimated using the full covariance matrix. The cell e.s.d.'s are taken into account individually in the estimation of e.s.d.'s in distances, angles and torsion angles; correlations between e.s.d.'s in cell parameters are only used when they are defined by crystal symmetry. An approximate (isotropic) treatment of cell e.s.d.'s is used for estimating e.s.d.'s involving l.s. planes.
Refinement. Refinement of *F*^2^ against ALL reflections. The weighted *R*-factor *wR* and goodness of fit *S* are based on *F*^2^, conventional *R*-factors *R* are based on *F*, with *F* set to zero for negative *F*^2^. The threshold expression of *F*^2^ > σ(*F*^2^) is used only for calculating *R*-factors(gt) *etc*. and is not relevant to the choice of reflections for refinement. *R*-factors based on *F*^2^ are statistically about twice as large as those based on *F*, and *R*- factors based on ALL data will be even larger.

### Fractional atomic coordinates and isotropic or equivalent isotropic displacement parameters (Å^2^)

**Table:**

	*x*	*y*	*z*	*U*_iso_*/*U*_eq_	
F1	0.53252 (12)	0.61885 (7)	0.61601 (11)	0.0484 (3)	
F2	0.39212 (17)	0.66320 (8)	0.45033 (11)	0.0610 (4)	
F3	0.31154 (14)	0.67824 (7)	0.62363 (12)	0.0544 (3)	
N1	0.22004 (15)	0.52452 (8)	0.43231 (12)	0.0284 (3)	
N2	0.16127 (15)	0.44751 (8)	0.45125 (11)	0.0269 (3)	
N3	0.07685 (16)	0.32384 (8)	0.35077 (13)	0.0311 (3)	
C1	0.30268 (18)	0.54413 (10)	0.54059 (14)	0.0288 (3)	
C2	0.29725 (19)	0.48203 (10)	0.62924 (14)	0.0310 (4)	
H2A	0.3470	0.4829	0.7128	0.037*	
C3	0.20539 (18)	0.42006 (10)	0.57018 (14)	0.0289 (3)	
C4	0.1508 (2)	0.34123 (11)	0.62036 (16)	0.0369 (4)	
H4A	0.1750	0.3423	0.7103	0.055*	
H4B	0.2054	0.2942	0.5892	0.055*	
H4C	0.0360	0.3353	0.5945	0.055*	
C5	0.07580 (17)	0.40647 (10)	0.34787 (13)	0.0266 (3)	
C6	0.0000	0.45258 (14)	0.2500	0.0265 (4)	
H6A	0.0000	0.5115	0.2500	0.032*	
C7	0.0000	0.28736 (15)	0.2500	0.0331 (5)	
H7A	0.0000	0.2284	0.2500	0.040*	
C8	0.3840 (2)	0.62608 (11)	0.55639 (15)	0.0342 (4)	

### Atomic displacement parameters (Å^2^)

**Table:**

	*U*^11^	*U*^22^	*U*^33^	*U*^12^	*U*^13^	*U*^23^
F1	0.0298 (5)	0.0524 (7)	0.0594 (7)	−0.0036 (5)	−0.0038 (5)	−0.0096 (5)
F2	0.0863 (10)	0.0544 (7)	0.0384 (6)	−0.0291 (7)	−0.0011 (6)	0.0066 (5)
F3	0.0461 (7)	0.0431 (6)	0.0754 (9)	0.0017 (5)	0.0146 (6)	−0.0206 (6)
N1	0.0275 (6)	0.0310 (7)	0.0259 (6)	−0.0002 (5)	0.0011 (5)	−0.0002 (5)
N2	0.0254 (6)	0.0302 (7)	0.0242 (6)	0.0013 (5)	0.0014 (5)	0.0006 (5)
N3	0.0300 (7)	0.0313 (7)	0.0311 (7)	0.0019 (5)	0.0023 (5)	0.0010 (6)
C1	0.0251 (7)	0.0353 (8)	0.0256 (7)	0.0034 (6)	0.0027 (6)	−0.0028 (6)
C2	0.0288 (8)	0.0393 (9)	0.0239 (7)	0.0063 (6)	0.0013 (6)	−0.0011 (6)
C3	0.0270 (7)	0.0350 (8)	0.0244 (7)	0.0070 (6)	0.0038 (6)	0.0024 (6)
C4	0.0432 (10)	0.0373 (9)	0.0296 (8)	0.0035 (7)	0.0044 (7)	0.0062 (7)
C5	0.0223 (7)	0.0324 (8)	0.0252 (7)	0.0012 (6)	0.0043 (6)	−0.0016 (6)
C6	0.0236 (10)	0.0292 (11)	0.0262 (10)	0.000	0.0028 (8)	0.000
C7	0.0341 (12)	0.0291 (11)	0.0352 (12)	0.000	0.0032 (9)	0.000
C8	0.0318 (8)	0.0394 (9)	0.0304 (8)	0.0008 (7)	0.0014 (6)	−0.0031 (7)

### Geometric parameters (Å, °)

**Table:**

F1—C8	1.3359 (19)	C2—C3	1.368 (2)
F2—C8	1.325 (2)	C2—H2A	0.9500
F3—C8	1.336 (2)	C3—C4	1.490 (2)
N1—C1	1.323 (2)	C4—H4A	0.9800
N1—N2	1.3668 (19)	C4—H4B	0.9800
N2—C3	1.377 (2)	C4—H4C	0.9800
N2—C5	1.414 (2)	C5—C6	1.3815 (19)
N3—C5	1.331 (2)	C6—C5^i^	1.3815 (19)
N3—C7	1.3319 (17)	C6—H6A	0.9500
C1—C2	1.404 (2)	C7—N3^i^	1.3319 (17)
C1—C8	1.489 (2)	C7—H7A	0.9500
			
C1—N1—N2	103.56 (13)	H4A—C4—H4C	109.5
N1—N2—C3	112.71 (12)	H4B—C4—H4C	109.5
N1—N2—C5	117.12 (12)	N3—C5—C6	123.83 (15)
C3—N2—C5	130.02 (14)	N3—C5—N2	116.59 (13)
C5—N3—C7	114.87 (15)	C6—C5—N2	119.57 (15)
N1—C1—C2	112.63 (15)	C5^i^—C6—C5	114.9 (2)
N1—C1—C8	119.21 (14)	C5^i^—C6—H6A	122.5
C2—C1—C8	128.13 (15)	C5—C6—H6A	122.5
C3—C2—C1	105.64 (14)	N3—C7—N3^i^	127.6 (2)
C3—C2—H2A	127.2	N3—C7—H7A	116.2
C1—C2—H2A	127.2	N3^i^—C7—H7A	116.2
C2—C3—N2	105.44 (14)	F2—C8—F1	107.01 (15)
C2—C3—C4	129.45 (15)	F2—C8—F3	107.49 (16)
N2—C3—C4	124.98 (15)	F1—C8—F3	105.65 (14)
C3—C4—H4A	109.5	F2—C8—C1	112.77 (14)
C3—C4—H4B	109.5	F1—C8—C1	111.59 (14)
H4A—C4—H4B	109.5	F3—C8—C1	111.93 (14)
C3—C4—H4C	109.5		
			
C1—N1—N2—C3	0.68 (16)	N1—N2—C5—N3	152.46 (14)
C1—N1—N2—C5	−175.36 (13)	C3—N2—C5—N3	−22.8 (2)
N2—N1—C1—C2	−0.63 (17)	N1—N2—C5—C6	−26.16 (18)
N2—N1—C1—C8	−179.10 (13)	C3—N2—C5—C6	158.62 (13)
N1—C1—C2—C3	0.37 (18)	N3—C5—C6—C5^i^	−0.48 (11)
C8—C1—C2—C3	178.67 (15)	N2—C5—C6—C5^i^	178.03 (15)
C1—C2—C3—N2	0.07 (17)	C5—N3—C7—N3^i^	−0.43 (10)
C1—C2—C3—C4	−175.86 (16)	N1—C1—C8—F2	−14.0 (2)
N1—N2—C3—C2	−0.47 (17)	C2—C1—C8—F2	167.76 (16)
C5—N2—C3—C2	174.92 (14)	N1—C1—C8—F1	−134.52 (15)
N1—N2—C3—C4	175.69 (14)	C2—C1—C8—F1	47.3 (2)
C5—N2—C3—C4	−8.9 (3)	N1—C1—C8—F3	107.31 (17)
C7—N3—C5—C6	0.9 (2)	C2—C1—C8—F3	−70.9 (2)
C7—N3—C5—N2	−177.66 (11)		

Symmetry codes: (i) −*x*, *y*, −*z*+1/2.
